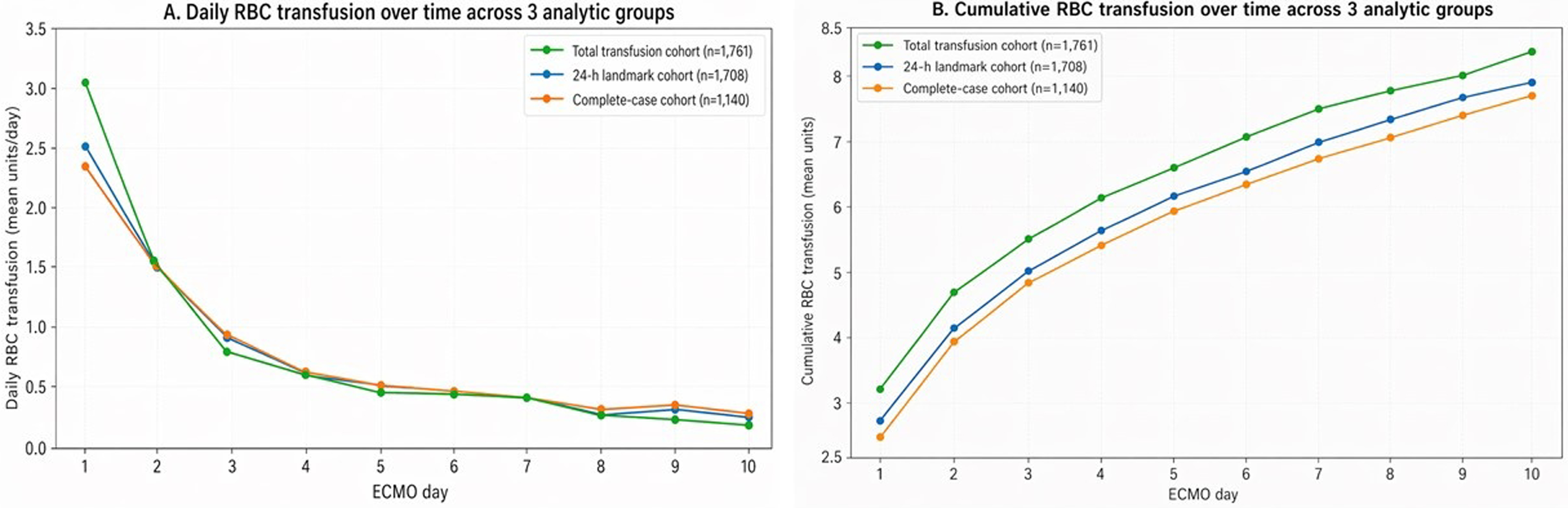# Early red blood cell transfusion burden, hemoglobin at first transfusion, and in-hospital mortality in patients supported with veno-arterial ECMO: a multicenter cohort study

**DOI:** 10.1016/j.aicoj.2026.100125

**Published:** 2026-07-25

**Authors:** Min Ho Ju, Seulgi Ju, In Seok Jeong, Hyoung Soo Kim, Yang Hyun Cho, Jae-Seung Jung, Jung-Hoon Shin, Sunghyun Sim, Hee Jung Kim

**Affiliations:** a Department of Thoracic and Cardiovascular Surgery and Research Institute for Convergence of Biomedical Science and Technology, Pusan National University Yangsan Hospital, Pusan National University School of Medicine, Yangsan, Republic of Korea; b Graduate School of AI Convergence Engineering, Changwon National University, 20 Changwondaehak-ro, Uichang-Gu, 51140 Changwon, South Korea; c Department of Thoracic and Cardiovascular Surgery, Chonnam National University Hospital, Gwangju, South Korea; d Department of Thoracic and Cardiovascular Surgery, Hallym University Sacred Heart Hospital, Hallym University Medical Center, Anyang-si, Gyeonggi-do 14068, Republic of Korea; e Department of Thoracic and Cardiovascular Surgery, Samsung Medical Center, School of Medicine, Sungkyunkwan University, Seoul, Republic of Korea; f Department of Thoracic and Cardiovascular Surgery, Korea University Anam Hospital, Korea University College of Medicine, Seoul, Republic of Korea; g Division of Cardiovascular Surgery, Department of Cardiovascular and Thoracic Surgery, Yonsei University College of Medicine, Seoul, Republic of Korea; h Department of AI Convergence Engineering, Changwon National University 20 Changwondaehak-ro, Uichang Gu, 51140 Changwon, South Korea

**Keywords:** Veno-Arterial extracorporeal membrane oxygenation, Red blood cell transfusion, Hemoglobin threshold, Transfusion strategy, In-Hospital mortality

## Abstract

**Background:**

Red blood cell (RBC) transfusion is common during venoarterial extracorporeal membrane oxygenation (VA-ECMO), but the prognostic relevance of early cumulative transfusion burden and hemoglobin (Hb) at transfusion initiation remains uncertain. We examined associations of ECMO day-1 RBC dose and, among transfused patients, first-transfusion Hb and transfusion-pattern phenotype with in-hospital mortality.

**Methods:**

Adults aged ≥18 years initiating peripheral VA-ECMO at six Korean centers between January 2018 and March 2025 were analyzed. The primary analysis used a 24 -h landmark cohort of patients alive and still receiving ECMO beyond 24 h. Day-1 RBC dose was categorized as 0, 1–2, 3–4, or ≥5 units and modeled per 2-unit increase. Adjusted risk ratios (RRs) were estimated using modified Poisson regression. Secondary analyses assessed first-transfusion Hb and consistent- versus variable threshold phenotypes among transfused complete cases.

**Results:**

Among 2,020 included patients, 1,708 entered the 24-h landmark cohort, and 1,140 complete cases comprised the adjusted primary analysis. Compared with no day-1 transfusion, adjusted RRs for in-hospital mortality were 1.20 (95% confidence interval [CI], 1.02–1.40) for 1–2 units, 1.37 (95% CI, 1.17–1.59) for 3–4 units, and 1.48 (95% CI, 1.28–1.71) for ≥5 units. Each 2-unit increase was associated with higher mortality (adjusted RR, 1.08; 95% CI, 1.05–1.11), with concordant findings over ECMO days 1–2 (adjusted RR, 1.06; 95% CI, 1.04–1.08). Among 1,043 transfused complete cases with qualifying pretransfusion Hb, each 1 g/dL decrease in first-transfusion Hb was associated with higher mortality (adjusted RR, 1.15; 95% CI, 1.10–1.20). The association was evident in the consistent-threshold phenotype (adjusted RR, 1.16; 95% CI, 1.10–1.22) but not in the variable-threshold phenotype (adjusted RR, 1.02; 95% CI, 0.92–1.12).

**Conclusions:**

In adults receiving peripheral VA-ECMO, early RBC transfusion burden showed a graded association with in-hospital mortality, whereas lower first-transfusion Hb was a prognostic marker chiefly within the consistent-threshold phenotype. These findings support dose- and threshold-aware prognostic assessment but do not establish causality or define an optimal transfusion threshold

## Highlights

• The 24-h landmark cohort included 1,708 adults from six VA-ECMO centers.
• Day-1 RBC dose showed a graded mortality association (RR 1.08 per 2 units).
• Exploratory analyses linked lower first-transfusion Hb to higher mortality.
• Most sensitivity analyses yielded directionally similar estimates.
• Findings are prognostic and do not establish an optimal transfusion threshold.

## Graphical abstract

**Figure:**
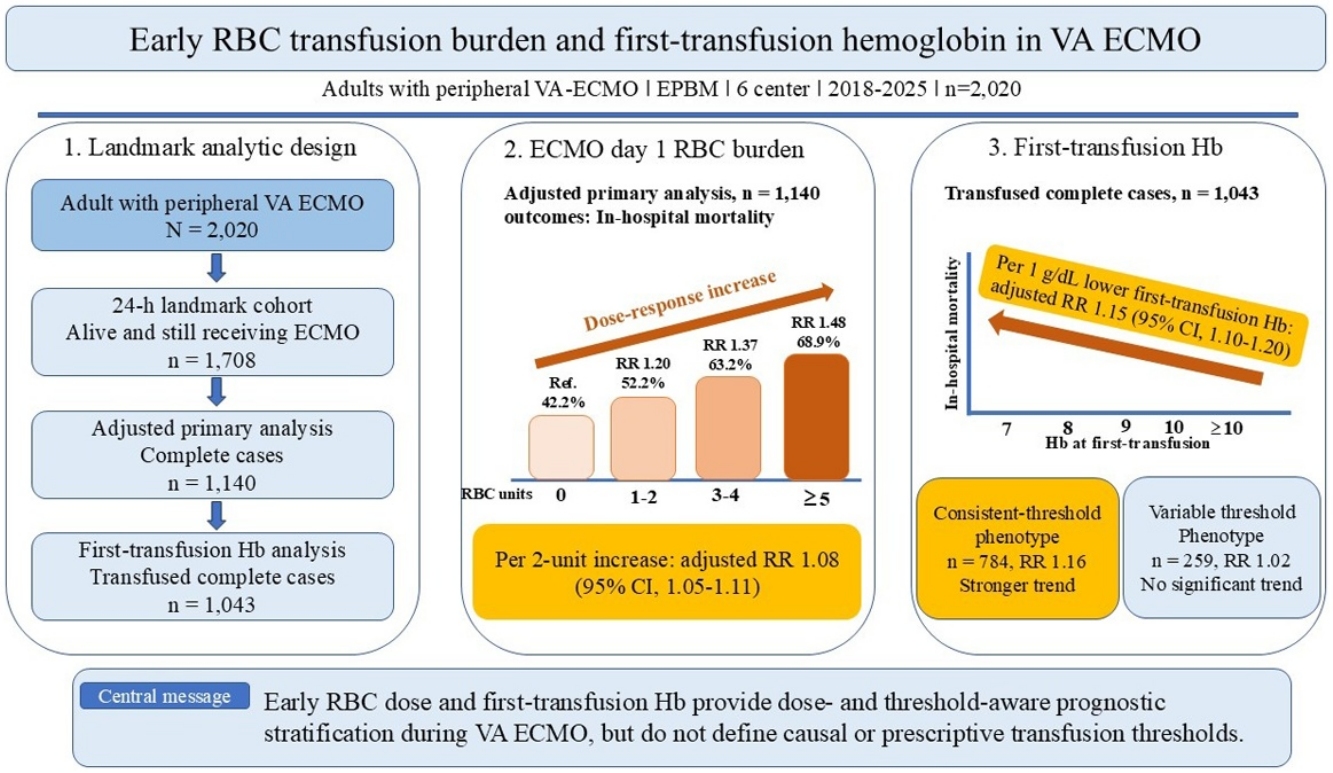

## Introduction

Red blood cell (RBC) transfusion is frequently required during extracorporeal membrane oxygenation (ECMO), particularly venoarterial ECMO (VA-ECMO). In addition to overt bleeding, patients supported with VA-ECMO are exposed to hemolysis, systemic anticoagulation, repeated invasive procedures, circuit-priming–related hemodilution, and ongoing volume shifts, all of which may increase transfusion requirements [[1], [2], [3]]. Yet the optimal hemoglobin (Hb) target during adult VA-ECMO remains uncertain, and transfusion practice varies substantially across centers [4,5]. Although contemporary transfusion guidelines recommend restrictive strategies (approximately 7 g/dL) for hemodynamically stable adults [6,7], these recommendations are not ECMO-specific and may not be directly applicable to the unique physiological conditions of VA-ECMO [8].

In venovenous ECMO, evidence has generally supported a restrictive transfusion approach. The international PROTECMO study demonstrated that the potential benefit of RBC transfusion was limited to patients with Hb levels below 7 g/dL, with no apparent advantage at higher thresholds [9]. In contrast, high-quality evidence in VA-ECMO remains scarce. A recent target-trial emulation using the international OBLEX dataset suggested a short-term benefit of liberal transfusion (Hb ≥9 g/dL) during the first few days after cannulation, but not beyond this early period [10]. Together, these data suggest that, in VA-ECMO, the clinical relevance of RBC transfusion may depend less on the mere occurrence of transfusion than on when transfusion is initiated, at what Hb level it occurs, and how transfusion exposure accumulates over time [11].

Because RBC transfusion is common and time-dependent during VA-ECMO, binary transfused versus non-transfused comparisons are vulnerable to confounding by indication and time-related bias. Clinically meaningful heterogeneity may therefore lie within the transfused population, where early transfusion burden, first-transfusion Hb, and transfusion pattern may provide more informative prognostic signals than transfusion exposure alone.

Using the multicenter Extracorporeal Patient Blood Management (EPBM) registry, a six-center Korean collaboration with standardized transfusion documentation, we aimed to characterize early RBC transfusion practice in adults supported with peripheral VA-ECMO and to examine the association between early cumulative RBC transfusion burden and in-hospital mortality. Among transfused patients, we secondarily evaluated first-transfusion Hb and transfusion-pattern phenotype as threshold-related prognostic markers.

## Methods

### Study design and population

**Fig. 1:**
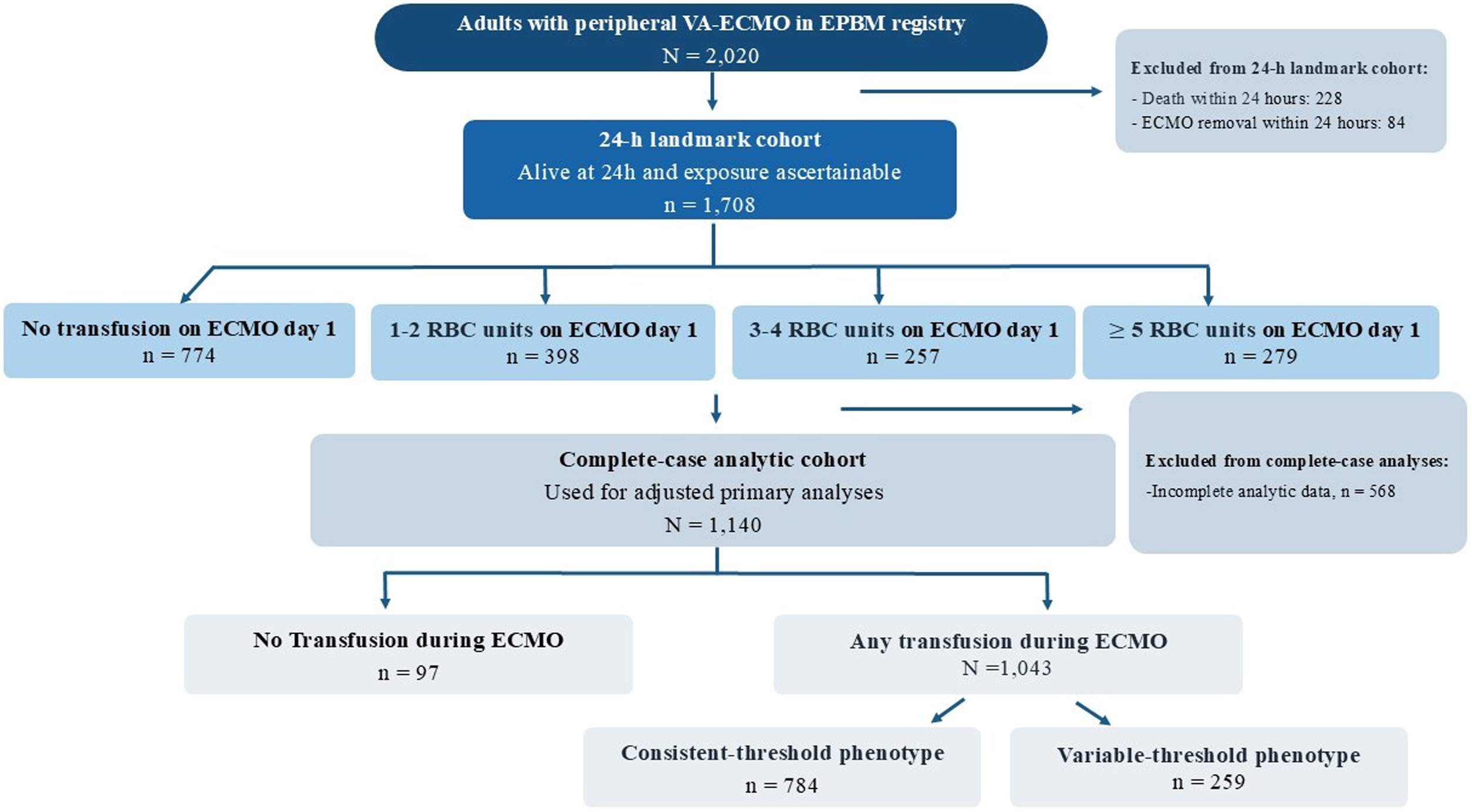
Flow diagram of the study population and analytic cohorts.

This multicenter cohort study used the EPBM registry, which includes adult VA-ECMO cases from six tertiary centers in South Korea. The registry prospectively collected data from 2022 onward and retrospectively incorporated cases from 2018 to 2021. Adults aged ≥18 years who initiated peripheral VA-ECMO between January 1, 2018, and March 20, 2025, were screened. Patients initially supported with central ECMO were excluded. Of 2,057 eligible registry patients, 37 with indeterminate in-hospital outcomes, including transfer while on ECMO or terminal discharge, were excluded, leaving 2,020 patients in the overall study population (Fig. 1).

Overall transfusion exposure during ECMO support was summarized descriptively as no transfusion versus ≥1 RBC transfusion (Supplementary Table S1). This comparison was not used as the primary inferential contrast because binary transfusion status is susceptible to confounding by indication and time-related bias.

The primary 24-h landmark cohort included patients who were alive and still receiving ECMO beyond 24 h after ECMO initiation (n = 1,708), excluding 228 patients who died and 84 who were decannulated within 24 h. Adjusted primary analyses were performed in 1,140 complete cases with available exposure, outcome, and prespecified covariates. Analyses of first-transfusion Hb and transfusion-pattern phenotype were restricted to 1,043 complete-case patients who received at least one RBC transfusion during ECMO support and had a qualifying pretransfusion Hb measurement (Fig. 1).

The study protocol was reviewed and approved by the Institutional Review Boards of all participating centers. Written informed consent was obtained from prospectively enrolled patients, whereas the requirement for consent was waived for retrospectively included patients because only de-identified data were used.

### Definition and subclassification of transfusion-related variables

RBC transfusions consisted of packed or leukocyte-reduced RBCs. Transfusion exposure was quantified as the cumulative number of RBC units administered during ECMO support. Because transfusion dates, but not clock times, were recorded, early exposure was analyzed by ECMO day. Accordingly, the day-1 exposure captured all RBC units administered during ECMO day 1 and did not allow separation of transfusions given immediately after cannulation from those administered later during the same calendar ECMO day. The primary early exposure was the total number of RBC units administered on ECMO day 1, and the expanded 48-h sensitivity exposure was the total number administered across ECMO days 1 and 2.

Pretransfusion Hb was defined as the closest available Hb measurement immediately preceding each RBC transfusion, using laboratory or point-of-care values. First-transfusion Hb was the pretransfusion Hb before the first RBC transfusion during ECMO support and was categorized as <7.0, 7.0–<8.0, 8.0–<9.0, 9.0–<10.0, and ≥10.0 g/dL. Because classification by first-transfusion Hb requires survival until transfusion, these analyses were restricted to the 24-h landmark cohort to reduce time-related bias [12,13].

Among transfused patients, transfusion-pattern phenotype was classified according to the distribution of recorded pretransfusion Hb categories across observed transfusion episodes. Patients whose transfusion episodes occurred within a single Hb category were classified as having a consistent-threshold phenotype, whereas those with transfusion episodes across two or more Hb categories were classified as having a variable-threshold phenotype. These classifications were based solely on the observed distribution of recorded pretransfusion Hb categories across transfusion episodes and were used as descriptive labels; information on clinician-intended thresholds or adherence to prespecified or center-specific transfusion protocols was not incorporated.

### Statistical analysis

Baseline and clinical characteristics were summarized using descriptive statistics. Continuous variables were presented as median [interquartile range], and categorical variables as n (%), based on available observations. Between-group differences were evaluated using both P values and standardized mean differences (SMDs). P values were calculated using the χ^2^ test or Fisher’s exact test for categorical variables and the Mann–Whitney U or Kruskal–Wallis test for continuous variables, as appropriate. For comparisons across four RBC-dose categories, SMD was defined as the maximum absolute pairwise SMD across all possible pairwise comparisons.

The primary outcome was in-hospital mortality. Within the 24-h landmark cohort, RBC units administered on ECMO day 1 were analyzed categorically as 0, 1–2, 3–4, and ≥5 units, with 0 units as the reference, and continuously per 2-unit increase.

Because in-hospital mortality was relatively frequent, adjusted risk ratios (RRs) were estimated using modified Poisson regression with robust, or sandwich, variance estimation. The 95% confidence intervals (CIs) were calculated using 2,000 nonparametric bootstrap resamples. For the first-transfusion Hb analysis, P values were also derived from the bootstrap distributions.

The primary model was adjusted for age, sex, BMI, pre-ECMO Hb, platelet count, lactate, creatinine, ECPR status, and postcardiotomy status, selected a priori based on clinical relevance. A separate severity-enhanced sensitivity model additionally included the pre-ECMO SOFA score.

Sensitivity analyses included a 48-h landmark analysis, in which early transfusion burden was redefined as cumulative RBC units administered during ECMO days 1–2, using the same adjusted modified Poisson regression model. Additional analyses were conducted after excluding postcardiotomy, heart transplantation, LVAD, or ECPR cases, and within a non-surgical VA-ECMO subgroup. Most analyses used modified Poisson regression with robust standard errors. A mixed-effects logistic regression model with center as a random effect was additionally fitted to account for center-level clustering, and results were reported as odds ratios (ORs) with 95% CIs. Fine–Gray models were fitted from the 24-h landmark, with in-hospital death as the event of interest and discharge alive as the competing event. ECMO removal was not considered a competing event because it did not preclude subsequent in-hospital death.

First-transfusion Hb was analyzed using predefined categories, with 8.0–<9.0 g/dL as the reference. Adjusted RRs were estimated using modified Poisson regression; pre-ECMO hemoglobin was not included to avoid overadjustment and collinearity. For trend analysis, Hb categories were assigned representative values of 6.5, 7.5, 8.5, 9.5, and 10.5 g/dL and modeled as a pseudo-continuous variable. Potential nonlinearity was assessed using restricted cubic splines with the same covariate adjustment set. Analyses were repeated within the consistent-threshold and variable-threshold phenotypes. To assess potential classification bias related to the number of observed transfusions, we performed a sensitivity analysis restricted to consistent-threshold patients who received ≥2 RBC units. Because exact transfusion timestamps were unavailable, distinct transfusion events could not be reliably reconstructed, and this analysis was considered a restriction sensitivity analysis rather than formal stratification by event count.

Exploratory analyses evaluated the interaction between ECMO day-1 RBC burden and first-transfusion Hb. A day-5 landmark analysis among patients remaining on ECMO for ≥120 h examined cumulative RBC burden during ECMO days 3–5, with adjustment for RBC burden during days 1–2 and the prespecified covariates.

Regression analyses were primarily performed in patients with complete data for the prespecified covariates; multiple imputation was used as a sensitivity analysis, and estimates were pooled using Rubin’s rules. All tests were two-sided, and P values <0.05 were considered statistically significant. Analyses were performed using Python version 3.10.

## Results

### Baseline characteristics and temporal distribution of RBC transfusion

Among 1,708 patients in the 24 -h landmark cohort, 774 (45.3%) received no RBC units on ECMO day 1, 398 (23.3%) received 1–2 units, 257 (15.0%) received 3–4 units, and 279 (16.3%) received ≥5 units (Table 1). Patients with greater day-1 RBC burden were generally characterized by older age, female sex, higher SOFA score, lower pre-ECMO Hb and platelet count, and more frequent postcardiotomy indication. Descriptively, mean daily RBC use was highest on ECMO day 1 and declined sharply by day 3, whereas cumulative exposure increased more gradually thereafter (Supplementary Fig. S1).

**Table 1:** Patient characteristics and clinical outcomes of the 24 -h landmark cohort according to cumulative RBC units administered on ECMO day 1.

*Variable*	Overall (N = 1,708)	0 units (n = 774)	1–2 units (n = 398)	3–4 units (n = 257)	≥5 units (n = 279)	Max pairwise SMD	P-value
*Demographics*							
Age, years	63.0 [53.0−72.0]	60.0 [52.0−69.0]	64.0 [55.0−73.0]	67.0 [57.0−75.0]	66.0 [55.5−74.0]	0.41	<0.001
BMI, kg/$m^{2}$	23.8 [21.6−26.3]	24.2 [22.1−26.6]	23.4 [21.3−25.7]	23.2 [21.2−26.1]	23.5 [21.1−26.1]	0.08	<0.001
Female sex	517 (30.3)	185 (23.9)	134 (33.7)	90 (35.0)	108 (38.7)	0.32	<0.001
*Medical history*							
Diabetes	543 (31.9)	214 (27.7)	123 (31.0)	109 (42.4)	97 (34.9)	0.31	<0.001
Hypertension	773 (45.4)	299 (38.8)	195 (49.1)	142 (55.3)	137 (49.3)	0.33	<0.001
Chronic kidney disease	114 (9.5)	29 (5.3)	39 (13.0)	20 (12.6)	26 (13.6)	0.28	<0.001
Myocardial infarction	137 (11.5)	62 (11.5)	39 (13.0)	18 (11.5)	18 (9.4)	0.11	0.697
Asthma/COPD	37 (3.2)	15 (2.8)	7 (2.4)	6 (3.9)	9 (4.8)	0.13	0.446
*Values before ECMO*							
SOFA-score	11.0 [8.0−13.0]	10.0 [8.0−13.0]	11.0 [8.0−13.0]	12.0 [9.0−14.5]	12.0 [10.0−15.0]	0.55	<0.001
Hemoglobin, g/dL	11.9 [9.7−14.1]	13.2 [11.4−15.1]	11.1 [9.3−13.3]	10.3 [8.6−12.6]	10.5 [8.5−12.6]	0.20	<0.001
Platelet count, $10^{9}$/L	186.0 [121.2−248.0]	200.0 [146.0−255.0]	181.5 [119.0−251.0]	164.0 [89.0−236.0]	168.5 [91.2−233.8]	0.17	<0.001
Lactate, mmol/L	5.6 [2.4−10.7]	5.6 [2.4−10.4]	4.2 [1.9−9.6]	5.9 [2.7−11.2]	7.5 [2.8−12.3]	0.43	<0.001
Creatinine, mg/dl	1.2 [0.9−1.8]	1.2 [0.9−1.6]	1.3 [0.9−1.8]	1.4 [1.0−2.0]	1.2 [0.9−2.0]	0.12	0.006
*ECMO characteristics*							
Duration, days	5.8 [3.0−10.8]	6.7 [3.5−13.0]	6.0 [3.2−11.0]	4.9 [2.7−8.1]	4.5 [2.3−7.9]	0.30	<0.001
Second run indicated	78 (4.6)	34 (4.4)	23 (5.8)	8 (3.1)	13 (4.7)	0.13	0.45
Distal perfusion	768 (45.0)	367 (47.4)	182 (46.0)	119 (46.3)	100 (36.0)	0.23	0.01
ECPR	639 (37.5)	272 (35.1)	143 (36.0)	108 (42.2)	116 (41.6)	0.14	0.086
*Indication ECMO*							
Acute Myocardial infarction	1,020 (67.9)	450 (65.1)	251 (69.9)	163 (74.8)	156 (66.4)	0.21	0.044
Post-cardiotomy	123 (7.2)	20 (2.6)	33 (8.3)	33 (12.8)	37 (13.3)	0.40	<0.001
Myocarditis	86 (5.7)	55 (8.0)	20 (5.6)	5 (2.3)	6 (2.6)	0.26	0.001
Pulmonary embolism	60 (4.0)	35 (5.1)	12 (3.3)	6 (2.8)	7 (3.0)	0.12	0.264
Others	337 (22.4)	151 (21.9)	76 (21.2)	44 (20.2)	66 (28.1)	0.18	0.144
*Purpose*							
Bridge to recovery	1,534 (90.0)	678 (87.6)	354 (89.6)	240 (93.4)	262 (93.9)	0.22	0.005
Bridge to advanced therapy	171 (10.0)	96 (12.4)	41 (10.4)	17 (6.6)	17 (6.1)	0.22	0.005
*Interventional treatment*							
PCI	242 (20.4)	127 (23.5)	58 (19.5)	29 (18.6)	28 (14.7)	0.22	0.061
Any cardiac surgery	178 (15.0)	82 (15.2)	45 (15.1)	20 (12.8)	31 (16.3)	0.10	0.837
-HT	87 (7.3)	44 (8.1)	28 (9.4)	10 (6.4)	5 (2.6)	0.28	0.033
-LVAD	16 (1.4)	10 (1.8)	3 (1.0)	2 (1.3)	1 (0.5)	0.12	0.523
-CABG	43 (3.6)	18 (3.3)	6 (2.0)	7 (4.5)	12 (6.3)	0.22	0.085
-Other cardiac surgery	32 (2.7)	10 (1.8)	8 (2.7)	1 (0.6)	13 (6.8)	0.33	<0.001
*Outcomes*							
ICU duration	13.0 [7.0−26.0]	14.0 [7.0−27.0]	15.0 [8.0−28.0]	12.0 [7.0−20.0]	11.0 [4.0−22.0]	0.18	<0.001
CRRT	864 (50.6)	329 (42.5)	196 (49.2)	145 (56.4)	194 (69.5)	0.54	<0.001
In hospital mortality	863 (50.5)	317 (41.0)	199 (50.0)	158 (61.5)	189 (67.7)	0.54	<0.001

SMD, standardized mean difference; BMI, body mass index; COPD, chronic obstructive pulmonary disease; SOFA, Sequential Organ Failure Assessment; ECMO, extracorporeal membrane oxygenation; ECPR, extracorporeal cardiopulmonary resuscitation; PCI, percutaneous coronary intervention; HT, heart transplantation; LVAD, left ventricular assist device; CABG, coronary artery bypass grafting; ICU, intensive care unit; CRRT, continuous renal replacement therapy.

*Values are presented as median [interquartile range] for continuous variables and n (%) for categorical variables. For both continuous and categorical variables, the number of non-missing observations is shown in parentheses. Percentages were calculated using non-missing observations.

Of the 1,708 patients in the 24 -h landmark cohort, 1,140 (66.7%) had complete data for all prespecified adjustment variables, whereas 568 (33.3%) were not included in from the primary adjusted analysis because at least one covariate was missing. Missingness was most frequent for lactate (448/1,708, 26.2%), followed by creatinine (240/1,708, 14.1%), platelet count (162/1,708, 9.5%), and pre-ECMO Hb (161/1,708, 9.4%). Compared with the complete-case cohort, excluded patients had a higher frequency of ECPR, higher lactate levels among those with available measurements, and numerically lower in-hospital mortality (Supplementary Table S2). Despite these differences, effect estimates from the multiple-imputation analyses were consistent in direction and similar in magnitude to those from the corresponding complete-case analyses (Supplementary Table S3-B).

### Cumulative RBC transfusion burden on ECMO day 1 and in-hospital mortality

**Fig. 2:**
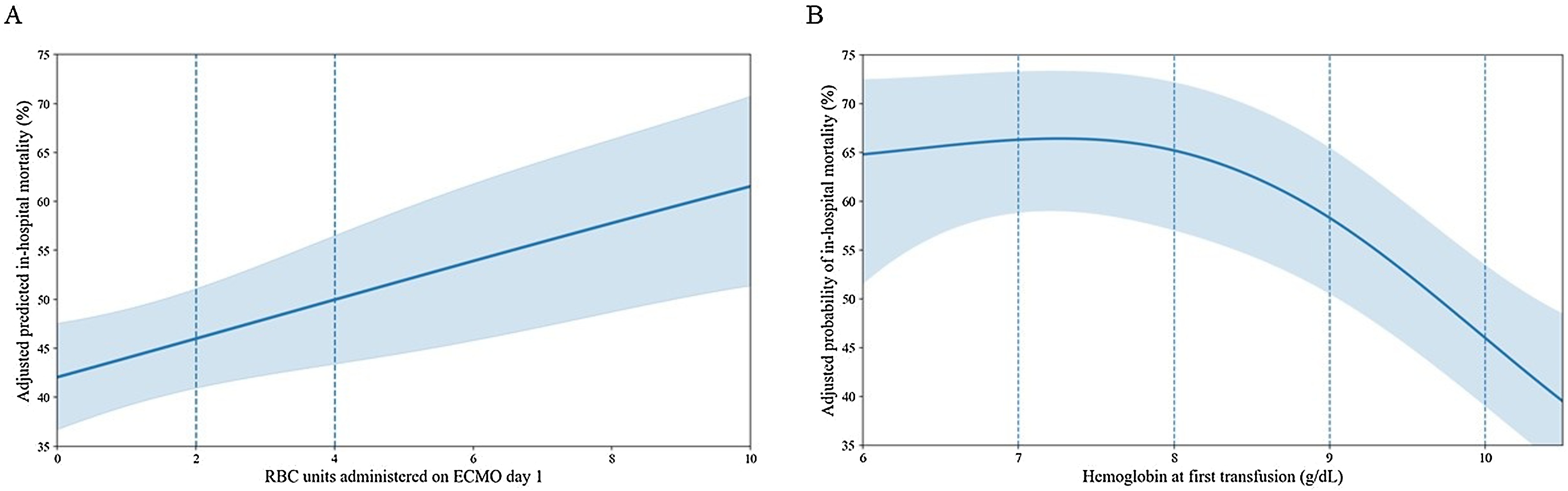
Adjusted associations of early RBC transfusion burden and hemoglobin at first transfusion with in-hospital mortality. (A) Adjusted dose-response curve for cumulative RBC units administered on ECMO day 1. (B) Adjusted association between hemoglobin at first RBC transfusion and in-hospital mortality. Solid lines indicate adjusted predicted probabilities, shaded areas indicate 95% confidence intervals, and vertical dashed lines indicate prespecified category cut-points.

In the complete-case cohort (n = 1,140), crude in-hospital mortality increased across ECMO day-1 RBC categories: 42.2% for 0 units, 52.2% for 1–2 units, 63.2% for 3–4 units, and 68.9% for ≥5 units (Table 2). Compared with no day-1 transfusion, adjusted RRs for in-hospital mortality were 1.20 (95% CI, 1.02–1.40; P = 0.021), 1.37 (95% CI, 1.17–1.59; P < 0.001), and 1.48 (95% CI, 1.28–1.71; P < 0.001) for the 1–2, 3–4, and ≥5unit groups, respectively. Modeled continuously, each 2-unit increase in day-1 RBC dose was associated with an 8% higher adjusted risk of in-hospital mortality (RR, 1.08; 95% CI, 1.05–1.11; P < 0.001), with a monotonic increase in the adjusted predicted probability of mortality across the observed dose range (Fig. 2A).

**Table 2:** Primary time-aware analysis of early RBC transfusion burden by ECMO day and in-hospital mortality.

Exposure	Patients	Deaths / N (%)	Adjusted RR (95% CI)	P value
RBC units administered on ECMO day 1 (n = 1,140)				
0 units	500	211/500 (42.2)	Reference	
1–2 units	278	145/278 (52.2)	1.20 (1.02–1.40)	0.021
3–4 units	185	117/185 (63.2)	1.37 (1.17–1.59)	<0.001
≥5 units	177	122/177 (68.9)	1.48 (1.28–1.71)	<0.001
Per 2-unit increase			1.08 (1.05–1.11)	<0.001
Cumulative RBC dose over ECMO day 1−2 (n = 1,056)				
0 units	338	133/338 (39.3)	Reference	
1–2 units	256	106/256 (41.4)	0.99 (0.81–1.21)	0.910
3–4 units	185	103/185 (55.7)	1.33 (1.09–1.61)	0.004
≥5 units	277	169/277 (61.0)	1.39 (1.17–1.65)	<0.001
Per 2-unit increase			1.06 (1.04–1.08)	<0.001

RBC, red blood cell; RR, risk ratio; CI, confidence interval.

*Adjusted RRs were estimated using modified Poisson regression with bootstrap 95% CIs and adjusted for age, sex, body mass index, pre-ECMO hemoglobin, platelet count, creatinine, lactate, extracorporeal cardiopulmonary resuscitation, and postcardiotomy status.

### Hemoglobin at first RBC transfusion and in-hospital mortality

Among 1,043 transfused complete-case patients with qualifying pretransfusion Hb measurements, lower first-transfusion Hb was associated with higher in-hospital mortality (Table 3). Compared with the 8.0–<9.0 g/dL reference group, first transfusion Hb <7.0 g/dL was associated with higher mortality (adjusted RR, 1.17; 95% CI, 1.01–1.35; P = 0.037), whereas first transfusion Hb ≥10.0 g/dL was associated with lower mortality (adjusted RR, 0.63; 95% CI, 0.51–0.78; P < 0.001). In the trend model based on assigned Hb-category values, each 1-g/dL decrease was associated with a 15% higher adjusted risk of mortality (RR, 1.15; 95% CI, 1.10–1.20; P < 0.001), and spline analysis showed no evidence of nonlinearity (P = 0.443; Fig. 2-B).

**Table 3:** First-transfusion hemoglobin and in-hospital mortality.

First-transfusion Hb in qualifying pretransfusion group (n = 1,043)	Patients	Deaths / N (%)	Adjusted RR (95% CI)	P-value
<7.0 g/dL	251	169/251 (67.3)	1.17 (1.01–1.35)	0.037
7.0–<8.0 g/dL	242	138/242 (57.0)	1.02 (0.87–1.20)	0.804
8.0–<9.0 g/dL	218	121/218 (55.5)	Reference	
9.0–<10.0 g/dL	126	55/126 (43.7)	0.82 (0.65–1.03)	0.089
≥10.0 g/dL	206	70/206 (34.0)	0.63 (0.51–0.78)	<0.001
Per 1 g/dL decrease			1.15 (1.10–1.20)	<0.001
Nonlinearity (spline)			P = 0.443	
First-transfusion Hb in consistent-threshold group (n = 784)				
<7.0 g/dL	146	96/146 (65.8)	1.31 (1.09–1.61)	0.003
7.0–<8.0 g/dL	169	93/169 (55.0)	1.12 (0.91–1.39)	0.281
8.0–<9.0 g/dL	157	78/157 (49.7)	Reference	
9.0–<10.0 g/dL	106	46/106 (43.4)	0.91 (0.68–1.19)	0.513
≥10.0 g/dL	206	70/206 (34.0)	0.71 (0.55–0.90)	0.007
Per 1 g/dL decrease			1.16 (1.10–1.22)	<0.001
Nonlinearity (spline)			P = 0.836	
First-transfusion Hb in variable threshold group (n = 259)				
<7.0 g/dL	105	73/105 (69.5)	0.92 (0.75–1.16)	0.438
7.0–<8.0 g/dL	73	45/73 (61.6)	0.86 (0.67–1.09)	0.219
8.0–<9.0 g/dL	61	43/61 (70.5)	Reference	
9.0–<10.0 g/dL	20	9/20 (45.0)	0.70 (0.36–1.05)	0.098
≥10.0 g/dL	0	–	–	–
Per 1 g/dL decrease			1.02 (0.92–1.12)	0.762
Nonlinearity (spline)			P = 0.579	

Hb, hemoglobin; RR, risk ratio; CI, confidence interval.

*The 8.0–<9.0 g/dL group was used as the reference. Consistent-threshold phenotype was defined as transfusions within a single Hb category; variable-threshold phenotype as transfusions across ≥2 Hb categories.

In phenotype-stratified analyses, the trend remained significant in the consistent-threshold group (n = 784; RR per 1-g/dL decrease, 1.16; 95% CI, 1.10–1.22; P < 0.001). The trend was not statistically significant in the variable-threshold group (n = 259; RR per 1-g/dL decrease, 1.02; 95% CI, 0.92–1.12; P = 0.762), which had a smaller sample size and included no patients with first-transfusion Hb ≥10.0 g/dL. In a sensitivity analysis restricted to consistent-threshold patients receiving ≥2 RBC units (n = 469), the trend remained statistically significant (RR per 1 g/dL decrease, 1.14; 95% CI, 1.08–1.21; P < 0.001), although category-specific estimates were less precise (Supplementary Table S4).

An exploratory multiplicative interaction was observed between ECMO day-1 RBC burden and first-transfusion Hb (adjusted RR for interaction, 0.96; 95% CI, 0.94–0.98; P < 0.001; Supplementary Table S5-A). Hb category-specific estimates are presented in Supplementary Table S5-B.

### Sensitivity analyses

Most sensitivity analyses yielded results in the same direction as the primary analysis. In the 48 -h landmark analysis, each 2-unit increase in cumulative RBC dose over ECMO days 1 and 2 was associated with higher in-hospital mortality (RR, 1.06; 95% CI, 1.04–1.08; P < 0.001). The day-1 association was also observed after excluding postcardiotomy, heart transplantation, or LVAD cases; after excluding ECPR cases; within the non-surgical VA-ECMO subgroup; and in the center-adjusted mixed-effects model (Supplementary Table S3-A). In the Fine–Gray analysis, each 2-unit increase in ECMO day-1 RBC dose was associated with a higher subdistribution hazard ratio (sHR) of in-hospital death (sHR, 1.16; 95% CI, 1.11–1.21; P < 0.001), with a graded association across RBC-dose categories (Supplementary Table S6).

Severity-enhanced analyses yielded directionally consistent but smaller estimates. Among 1,110 patients in the SOFA-complete cohort, each 2-unit increase in ECMO day-1 RBC dose remained associated with higher in-hospital mortality after additional adjustment for pre-ECMO SOFA score (RR, 1.05; 95% CI, 1.02–1.08; P < 0.001). A complementary multiple-imputation analysis of the 598 patients not included in the SOFA-complete cohort yielded a similar estimate (RR, 1.04; 95% CI, 1.02–1.06; P < 0.001), with broadly similar findings in additional sensitivity analyses (Supplementary Table S3-B).

In an exploratory day-5 landmark analysis among patients who remained on ECMO support for at least 120 h, greater RBC transfusion burden during ECMO days 3–5 was associated with higher in-hospital mortality after adjustment for early RBC burden and prespecified covariates (Supplementary Table S7). A model including the overall day-5 landmark cohort and adjusting for early RBC burden yielded a similar result.

## Discussion

In this multicenter cohort of adults supported with peripheral VA-ECMO, greater early RBC transfusion burden was associated with higher in-hospital mortality. Specifically, cumulative RBC dose on ECMO day 1 demonstrated a graded association with mortality across predefined dose categories and when modeled as a continuous exposure. This relationship was directionally similar in the 48 -h analysis and across most sensitivity analyses, including the competing-risk analysis. Descriptive temporal trajectories showed that RBC transfusion was concentrated early after ECMO initiation. In the secondary analysis, lower Hb at the first RBC transfusion was also associated with higher mortality, with a more apparent signal among patients managed with a consistent-threshold transfusion pattern. Prior studies in general critical care populations have shown that anemia and RBC transfusion are common and often associated with illness severity and adverse outcomes [14,15], while ECMO-specific studies have shown that bleeding and RBC transfusion are common during ECMO and associated with adverse outcomes [1,3,16]. Taken together, our findings extend the existing literature by shifting the analytic focus from binary transfusion exposure to early transfusion intensity and threshold-related practice patterns.

Early cumulative RBC burden provides a more granular characterization of transfusion exposure than binary transfusion status because transfusion exposure accumulates during a rapidly evolving course of shock, bleeding, and procedural recovery [17,18]. This focus is consistent with recent ECMO transfusion literature emphasizing transfusion thresholds, cumulative exposure, and practice heterogeneity [9,10]. Larger early RBC requirements may reflect bleeding, procedural or postcardiotomy complexity, hemodynamic instability, hemolysis, coagulopathy, and limited physiologic reserve rather than the effect of transfusion alone [19,20]. ECMO-related blood trauma and the uncertain relationship between increased Hb concentration and tissue oxygenation further support cautious interpretation [4,21,22]. Accordingly, the observed graded association should be interpreted as prognostic rather than causal and may, at least in part, reflect the clinical conditions that prompted transfusion.

Findings regarding Hb at the first RBC transfusion should be interpreted as exploratory and prognostic rather than prescriptive. Importantly, the lower mortality observed among patients first transfused at Hb ≥10.0 g/dL, compared with the 8.0–<9.0 g/dL reference group, should not be interpreted as evidence that a liberal transfusion threshold is beneficial. First-transfusion Hb was an observed pretransfusion measurement rather than a randomized or protocolized treatment threshold, and patients transfused at Hb ≥10.0 g/dL may have represented a clinically selected subgroup treated under different procedural, bleeding, or hemodynamic circumstances. Conversely, lower first-transfusion Hb may reflect ongoing blood loss, physiologic instability, or a more adverse clinical course. Because the timing, indications, and clinical context of the first transfusion were incompletely captured, patient selection, context-specific transfusion practices, and residual confounding may account for this apparently favorable association. Therefore, this finding does not imply that initiating transfusion at a higher Hb threshold would improve outcomes. Restrictive transfusion strategies are well supported in general critical care and cardiac surgery populations, but their applicability to VA-ECMO remains uncertain [6,7,23,24]. Current ECMO-specific evidence remains limited by heterogeneity in patient populations, transfusion indications, and clinical triggers [5]. Recent target-trial emulation data further underscore this uncertainty [10]. Accordingly, first-transfusion Hb should be viewed as an exploratory prognostic correlate rather than a therapeutic target.

In phenotype-stratified analyses, the association between lower first-transfusion Hb and mortality was observed in the consistent-threshold group but not in the variable-threshold group. Although descriptively informative, these phenotypes were derived from observed Hb-category patterns and should not be regarded as proxies for prespecified or consistently implemented transfusion strategies. Because phenotype assignment depended on the number and distribution of recorded transfusion episodes, it may have been influenced by transfusion frequency, exposure duration, and evolving clinical status. Accordingly, the between-phenotype difference should be considered hypothesis-generating within the broader context of cardiogenic shock heterogeneity, in which outcomes are influenced by dynamic changes during mechanical circulatory support [[25], [26], [27]].

The strengths of this study include its multicenter design, relatively large cohort of adults receiving peripheral VA-ECMO, and detailed documentation of transfusion and ECMO-related variables. The dose-resolved landmark approach allowed early transfusion exposure to be evaluated beyond a simple binary comparison. Given the substantial clinical heterogeneity of VA-ECMO, we performed several complementary sensitivity analyses, including alternative exposure windows, clinically relevant subgroup exclusions, center-adjusted models, additional SOFA adjustment, multiple imputation, and competing-risk analysis. Although the primary adjusted analyses were restricted to patients with complete covariate data, additional analyses using imputed datasets were performed to assess the robustness of the findings.

Several limitations should be acknowledged. First, as an observational registry-based study, the analysis cannot establish causality, and residual confounding, particularly confounding by indication, remains possible. Although the primary model included several clinically relevant markers of illness severity and additional adjustment for pre-ECMO SOFA score yielded a directionally consistent but smaller association, unmeasured severity, bleeding, and procedural factors may still partly explain the observed associations. Second, exact transfusion timestamps and detailed clinical triggers, including bleeding severity, transfusion indication, anticoagulation intensity, circuit events, and clinician-level decision-making, were not fully captured. Because transfusion exposure was aggregated at the ECMO-day level, we could not distinguish transfusions administered during the immediate peri-cannulation period or first several hours from those administered later on ECMO day 1, or determine the within-day sequence of bleeding, clinical deterioration, and transfusion. Therefore, “early transfusion” in this study refers to cumulative transfusion exposure during ECMO day 1, whereas “very early transfusion,” such as transfusion during cannulation or within the first several hours after ECMO initiation, could not be separately defined or evaluated. Accordingly, day-1 RBC dose should be interpreted as an aggregate measure of early transfusion burden rather than a precisely timed treatment exposure. Furthermore, because first transfusion could occur after the 24-h landmark, residual time-related selection may remain in analyses involving first-transfusion Hb. Third, adjusted analyses were restricted to the complete-case cohort. Patients excluded because of missing covariate data differed from included patients in several severity- and treatment-related characteristics, suggesting that missingness may not have been completely random. Therefore, selection bias cannot be excluded, although full-cohort multiple-imputation analyses yielded broadly similar estimates. Finally, transfusion-pattern classification was inherently dependent on the number of observed transfusions, because patients with greater transfusion exposure had more opportunities to cross Hb categories and be classified as having a variable-threshold pattern. Although analyses restricted to patients receiving ≥2 RBC units reduced the influence of patients with limited transfusion exposure, residual classification bias related to transfusion frequency, exposure duration, and illness severity could not be excluded.

Taken together, these findings should not be interpreted as defining an optimal Hb threshold or establishing a causal effect of RBC transfusion in VA-ECMO. Instead, early RBC transfusion burden and Hb at first transfusion may serve as prognostic correlates of clinical instability and transfusion complexity. Future prospective studies incorporating time-stamped transfusion data, standardized bleeding assessment, physiologic transfusion triggers, and protocolized transfusion strategies are needed to determine whether specific transfusion strategies can improve outcomes in patients receiving VA-ECMO.

## Conclusion

In adults receiving peripheral VA-ECMO, greater RBC transfusion burden on ECMO day 1 and lower Hb at first transfusion were associated with higher in-hospital mortality, with the latter association being most apparent in the consistent-threshold phenotype. These associations should be interpreted as prognostic rather than causal and may reflect bleeding, procedural complexity, hemodynamic instability, and overall illness severity. The observed dose-response relationship does not establish that RBC transfusion itself causes mortality. Early transfusion burden and first-transfusion Hb may therefore be useful for risk characterization but should not be used to define a transfusion threshold.

## Authors’ contributions

Min Ho Ju: Conceptualization; Methodology; Formal analysis; Investigation; Data curation; Writing – original draft; Writing – review & editing; Visualization; Supervision.

Seulgi Ju: Conceptualization; Methodology; Formal analysis; Investigation; Data curation; Writing – original draft; Writing – review & editing.

In Seok Jeong: Methodology; Formal analysis; Validation; Writing – review & editing.

Hyoung Soo Kim: Investigation; Resources; Data curation; Writing – review & editing.

Yang Hyun Cho: Investigation; Resources; Writing – review & editing.

Jae-Seung Jung: Investigation; Resources; Writing – review & editing.

Jung-Hoon Shin: Investigation; Resources; Writing – review & editing.

Sunghyun Sim: Conceptualization; Supervision; Software; Formal analysis; Visualization; Writing – review & editing

Hee Jung Kim: Conceptualization; Supervision; Project administration; Writing – review & editing.

All authors approved the final version of the manuscript.

## Ethics approval and consent to participate

This study was conducted in accordance with the Declaration of Helsinki and was approved by the Institutional Review Boards of all participating centers. Written informed consent was obtained from prospectively enrolled patients. For retrospectively included patients, the requirement for informed consent was waived because only de-identified data were analyzed.

## Declaration of Generative AI and AI-assisted technologies in the writing process

During the preparation of this manuscript, the authors used ChatGPT (OpenAI, San Francisco, CA, USA) to assist with language editing and improvement of clarity and academic English expression. The generative AI tool was not used for study design, data analysis, statistical modeling, or interpretation of the results. All scientific content, analyses, and conclusions were developed independently by the authors. The authors take full responsibility for the integrity, accuracy, and originality of the work.

## Data availability

The data that has been used is confidential.

## Declaration of competing interest

On behalf of all authors, the corresponding author states that there is no conflict of interest.

## Figures and Tables

**Figure:**