# Supplementary material for: Early red blood cell transfusion burden, hemoglobin at first transfusion, and in-hospital mortality in patients supported with veno-arterial ECMO: a multicenter cohort study
Source: Ann Intensive Care. 2026 Jul 25;16:100125. doi: 10.1016/j.aicoj.2026.100125 (PMC13452410; doi:10.1016/j.aicoj.2026.100125)
Supplement: Supplementary file 1 [file mmc1.docx]

**Supplementary table 1. Descriptive baseline characteristics according to transfusion exposure**

| Variable | Non-transfusion  (n=259) | Transfusion  (n=1,761) | *p*-value |
| --- | --- | --- | --- |
| *Demographics* |  |  |  |
| Age, years | 62 [52–70] | 64 [54–73] | 0.013 |
| BMI, kg/$\boldsymbol{m}^{\boldsymbol{2}}$ | 24.8 [22.5–27.3] | 23.6 [21.3–26.1] | < 0.001 |
| Female sex | 54 (20.8%) | 567 (32.2%) | < 0.001 |
| *Medical history* |  |  |  |
| Diabetes | 73 (29.0%) | 562 (32.0%) | 0.347 |
| Hypertension | 98 (39.4%) | 835 (47.6%) | 0.017 |
| Chronic kidney disease | 9 (5.4%) | 130 (10.3%) | 0.050 |
| Myocardial infarction | 23 (13.9%) | 141 (11.2%) | 0.301 |
| Asthma/COPD | 4 (2.5%) | 42 (3.4%) | 0.646 |
| *Values before ECMO* |  |  |  |
| SOFA-score | 12 [9–13] | 11 [9–14] | 0.751 |
| Hemoglobin, g/dL | 13.4 [11.5–15.0] | 11.5 [9.5–13.7] | < 0.001 |
| Platelet count, ${10}^{9}$/L | 188 [140.0–247] | 181 [111–246] | 0.091 |
| Lactate, $mmol/L$ | 7.7 [3.7–12.3] | 5.7 [2.3–11.0] | <0.001 |
| Creatinine, m$g/dl$ | 1.3 [1.0–1.6] | 1.2 [0.9–1.8] | 0.627 |
| *ECMO characteristics* |  |  |  |
| Duration, days | 2.2 [0.2–5.2] | 5.1 [2.2–10.1] | < 0.001 |
| Second run indicated | 3 (1.2%) | 81 (4.6%) | 0.007 |
| Distal perfusion | 95 (36.7%) | 728 (41.3%) | 0.166 |
| ECPR | 133 (51.4%) | 685 (38.9%) | < 0.001 |
| *Indication ECMO* |  |  | 0.018 |
| Acute Myocardial infarction | 145 (56.0%) | 945 (53.7%) |  |
| Post-cardiotomy | 7 (2.7%) | 142 (8.1%) |  |
| Myocarditis | 8 (3.1%) | 74 (4.2%) |  |
| Pulmonary embolism | 6 (2.3%) | 55 (3.1%) |  |
| Others | 93 (35.9%) | 545 (30.9%) |  |
| *Purpose* |  |  | 0.002 |
| Bridge to recovery | 248 (95.8%) | 1,573(89.3%) |  |
| Bridge to advanced therapy | 11 (4.2%) | 184 (10.4%) |  |
| *Interventional treatment* |  |  | <0.001 |
| PCI | 44 (25.1%) | 239 (19.2%) |  |
| Any cardiac surgery | 9 (5.1%) | 189 (15.1%) |  |
| -HT | 4 (2.3%) | 83 (6.7%) |  |
| -LVAD | 1 (0.6%) | 15 (1.2%) |  |
| -CABG | 3 (1.7%) | 48 (3.8%) |  |
| -Other cardiac surgery | 1 (0.6%) | 43 (3.4%) |  |
| *Outcomes* |  |  |  |
| ICU duration | 6.0 [3.0-13.0] | 13.0 [6.0-25.0] | <0.001 |
| CRRT | 62 (23.9%) | 903 (51.3%) | <0.001 |
| In hospital mortality | 144 (55.6%) | 960 (54.5%) | 0.789 |

BMI, body mass index; COPD, chronic obstructive pulmonary disease; SOFA, Sequential Organ Failure Assessment; ECMO, extracorporeal membrane oxygenation; ECPR, extracorporeal cardiopulmonary resuscitation; PCI, percutaneous coronary intervention; HT, heart transplantation; LVAD, left ventricular assist device; CABG, coronary artery bypass grafting; ICU, intensive care unit; CRRT, continuous renal replacement therapy.
