# Supplementary material for: Early red blood cell transfusion burden, hemoglobin at first transfusion, and in-hospital mortality in patients supported with veno-arterial ECMO: a multicenter cohort study
Source: Ann Intensive Care. 2026 Jul 25;16:100125. doi: 10.1016/j.aicoj.2026.100125 (PMC13452410; doi:10.1016/j.aicoj.2026.100125)
Supplement: Supplementary file 2 [file mmc2.docx]

**Supplementary Table 2. Comparison of patients included versus excluded from the complete-case analytic cohort**

| *Variable* | Overall  (N=1,708) | complete- cohort  (n=1,140) | Excluded cohort  (n=568) | SMD | P-value |
| --- | --- | --- | --- | --- | --- |
| *Demographics* |  |  |  |  |  |
| Age, years | 63.0 [53.0-72.0] | 63.0 [53.0-72.0] | 63.0 [54.0-72.0] (568/568) | 0.016 | 0.720 |
| BMI, kg/$\boldsymbol{m}^{\boldsymbol{2}}$ | 23.8 [21.6-26.3] | 23.8 [21.6-26.3] | 23.8 [21.6-26.2] (557/568) | 0.058 | 0.726 |
| Female sex | 517 (30.3) | 345 (30.3) | 172 (30.3) (568/568) | 0.000 | 1.000 |
| *Medical history* |  |  |  |  |  |
| Diabetes | 543 (31.9) | 374 (32.8) | 169 (30.0) (564/568) | 0.061 | 0.259 |
| Hypertension | 773 (45.4) | 534 (46.9) | 239 (42.4) (564/568) | 0.092 | 0.085 |
| Chronic kidney disease | 114 (9.5) | 77 (9.7) | 37 (9.2) (400/568) | 0.015 | 0.885 |
| Myocardial infarction | 137 (11.5) | 74 (9.4) | 63 (15.8) (400/568) | 0.194 | 0.002 |
| Asthma/COPD | 37 (3.2) | 18 (2.3) | 19 (4.9) (390/568) | 0.136 | 0.033 |
| *Values before ECMO* |  |  |  |  |  |
| SOFA-score | 11.0 [8.0-13.0] | 11.0 [9.0-13.0] | 10.0 [8.0-13.0] (497/568) | 0.227 | <0.001 |
| Hemoglobin, g/dL | 11.9 [9.7-14.1] | 11.7 [9.5-14.0] | 12.3 [10.3-14.1] (407/568) | 0.006 | 0.015 |
| Platelet count, $\boldsymbol{10}^{\boldsymbol{9}}$/L | 186.0 [121.2-248.0] | 180.5 [114.0-246.0] | 196.0 [131.2-253.0] (406/568) | 0.102 | 0.036 |
| Lactate, $\boldsymbol{mmol/L}$ | 5.6 [2.4-10.7] | 5.1 [2.3-10.4] | 9.3 [6.3-13.1] (120/568) | 0.469 | <0.001 |
| Creatinine, m$\boldsymbol{g/dl}$ | 1.2 [0.9-1.8] | 1.3 [0.9-1.8] | 1.1 [0.9-1.5] (328/568) | 0.108 | <0.001 |
| *ECMO characteristics* |  |  |  |  |  |
| Duration, days | 5.8 [3.0-10.8] | 6.0 [3.2-11.1] | 5.2 [2.8-9.9] (568/568) | 0.104 | 0.010 |
| Second run indicated | 78 (4.6) | 57 (5.0) | 21 (3.7) (568/568) | 0.065 | 0.264 |
| Distal perfusion | 768 (45.0) | 478 (42.0) | 290 (51.2) (566/568) | 0.187 | <0.001 |
| ECPR | 639 (37.5) | 387 (33.9) | 252 (44.5) (566/568) | 0.218 | <0.001 |
| *Indication ECMO* |  |  |  |  |  |
| Acute Myocardial infarction | 1,020 (67.9) | 668 (67.3) | 352 (69.0) (510/568) | 0.038 | 0.529 |
| Post-cardiotomy | 123 (7.2) | 88 (7.7) | 35 (6.2) (568/568) | 0.061 | 0.283 |
| Myocarditis | 86 (5.7) | 57 (5.7) | 29 (5.7) (510/568) | 0.002 | 1.000 |
| Pulmonary embolism | 60 (4.0) | 42 (4.2) | 18 (3.5) (510/568) | 0.036 | 0.605 |
| Others | 337 (22.4) | 226 (22.8) | 111 (21.8) (510/568) | 0.024 | 0.710 |
| *Purpose* |  |  |  |  |  |
| Bridge to recovery | 1,534 (90.0) | 1,025 (90.1) | 509 (89.8) (567/568) | 0.010 | 0.914 |
| Bridge to advanced therapy | 171 (10.0) | 113 (9.9) | 58 (10.2) (567/568) | 0.010 | 0.914 |
| *Interventional treatment* |  |  |  |  |  |
| PCI | 242 (20.4) | 122 (15.6) | 120 (29.9) | 0.348 | <0.001 |
| Any cardiac surgery | 178 (15.0) | 115 (14.7) | 63 (15.7) | 0.029 | 0.697 |
| -HT | 87 (7.3) | 54 (6.9) | 33 (8.2) | 0.051 | 0.471 |
| -LVAD | 16 (1.4) | 8 (1.0) | 8 (2.0) | 0.080 | 0.267 |
| -CABG | 43 (3.6) | 30 (3.8) | 13 (3.2) | 0.032 | 0.730 |
| -Other cardiac surgery | 32 (2.7) | 23 (2.9) | 9 (2.2) | 0.043 | 0.615 |
| *Outcomes* |  |  |  |  |  |
| ICU duration | 13.0 [7.0-26.0] | 14.0 [8.0-28.0] | 11.0 [6.0-21.0] (558/568) | 0.152 | <0.001 |
| CRRT | 864 (50.6) | 601 (52.7) | 263 (46.3) (568/568) | 0.129 | 0.014 |
| In hospital mortality | 863 (50.5) | 595 (52.2) | 268 (47.2) (568/568) | 0.100 | 0.057 |

SMD, standardized mean difference; BMI, body mass index; COPD, chronic obstructive pulmonary disease; SOFA, Sequential Organ Failure Assessment; ECMO, extracorporeal membrane oxygenation; ECPR, extracorporeal cardiopulmonary resuscitation; PCI, percutaneous coronary intervention; HT, heart transplantation; LVAD, left ventricular assist device; CABG, coronary artery bypass grafting; ICU, intensive care unit; CRRT, continuous renal replacement therapy.

Values are presented as median [IQR] or n/N (%). Percentages were calculated among patients with non-missing data.
