# Supplementary material for: Early red blood cell transfusion burden, hemoglobin at first transfusion, and in-hospital mortality in patients supported with veno-arterial ECMO: a multicenter cohort study
Source: Ann Intensive Care. 2026 Jul 25;16:100125. doi: 10.1016/j.aicoj.2026.100125 (PMC13452410; doi:10.1016/j.aicoj.2026.100125)
Supplement: Supplementary file 3 [file mmc3.docx]

**Supplementary Table 3-A. Prespecified sensitivity analyses using the original covariate set**

| Sensitivity analysis | Exposure metric | Original complete-case cohort | | | Excluded cohort with MI | | | | | Full cohort with MI | | | |
| --- | --- | --- | --- | --- | --- | --- | --- | --- | --- | --- | --- | --- | --- |
|  |  | **n** | **Effect estimate** | **P-value** | | **n** | **Effect estimate** | | **P-value** | | **n** | **Effect estimate** | **P-value** |
| Primary model: 24-h landmark cohort | Per 2-unit increase in RBC dose on ECMO day 1 | 1,140 | RR 1.08 (1.05–1.11) | <0.001 | | 568 | RR 1.06 (1.04–1.08) | | <0.001 | | 1,708 | RR 1.06 (1.04–1.08) | <0.001 |
| 48-h landmark cohort | Per 2-unit increase in cumulative RBC dose over the first 2 ECMO days | 1,056 | RR 1.06 (1.04–1.08) | <0.001 | | 527 | RR 0.98 (0.75–1.28) | | 0.89 | | 1,583 | RR 1.06 (1.04–1.07) | <0.001 |
| Excluding postcardiotomy / HT / LVAD | Per 2-unit increase in RBC dose on ECMO day 1 | 1,017 | RR 1.08 (1.05–1.11) | <0.001 | | 503 | RR 1.05 (1.03–1.07) | | <0.001 | | 1,520 | RR 1.06 (1.04–1.08) | <0.001 |
| Excluding ECPR | Per 2-unit increase in RBC dose on ECMO day 1 | 753 | RR 1.09 (1.06–1.13) | <0.001 | | 314 | RR 0.86 (0.45–1.67) | | 0.662 | | 1,067 | RR 1.07 (1.05–1.10) | <0.001 |
| Non-surgical VA-ECMO only | Per 2-unit increase in RBC dose on ECMO day 1 | 478 | RR 1.09 (1.05–1.12) | <0.001 | | 177 | RR 1.04 (1.02–1.07) | | 0.002 | | 655 | RR 1.05 (1.03–1.08) | <0.001 |
| Center-adjusted mixed-effects model | Per 2-unit increase in RBC dose on ECMO day 1 | 1,140 | OR 1.27 (1.18–1.36) | <0.001 | | 568 | | OR 1.26  (1.15–1.37) | <0.001 | | 1,708 | OR 1.25 (1.19–1.32) | <0.001 |

RBC, red blood cell; RR, risk ratio; OR, odds ratio; CI, confidence interval; ECMO, extracorporeal membrane oxygenation; VA, veno-arterial; ECPR, extracorporeal cardiopulmonary resuscitation; HT, heart transplantation; LVAD, left ventricular assist device.

* Risk ratios (RR) were estimated using modified Poisson regression models, adjusted for Age, Sex, BMI, Pre-ECMO hemoglobin, Platelet count, Creatinine, Lactate, ECPR, and Postcardiotomy. In the analysis excluding ECPR, the ECPR variable was omitted from the adjustment set. The center-adjusted mixed-effects model was performed using a mixed-effects logistic model with center included as a random effect, and the results are presented as odds ratios (OR) with 95% confidence intervals. The complete cohort included patients with complete data for the original prespecified covariates. Patients not included in the complete cohort had missing data in at least one original covariate; for the excluded subgroup and the full cohort, missing covariates were imputed using multiple imputation, and estimates were pooled using Rubin’s rules. Risk ratios were estimated using modified Poisson regression with robust variance estimation and adjusted for age, sex, body mass index, pre-ECMO hemoglobin, platelet count, creatinine, lactate, ECPR status, and postcardiotomy status. In the analysis excluding ECPR, the ECPR variable was omitted from the adjustment set. The center-adjusted mixed-effects model included center as a random effect and is presented as an odds ratio. Excluded-cohort-only MI estimates with wide confidence intervals should be interpreted cautiously because of limited precision.

**Supplementary Table 3-B. Severity-enhanced sensitivity analyses with additional SOFA adjustment and multiple imputation**

| Sensitivity analysis | Exposure metric | SOFA-complete cohort | | | Excluded cohort with MI | | |
| --- | --- | --- | --- | --- | --- | --- | --- |
|  |  | **n** | **Effect estimate** | **P-value** | **n** | **Effect estimate** | **P-value** |
| Primary model: 24-h landmark cohort | Per 2-unit increase in RBC dose on ECMO day 1 | 1,110 | RR 1.05 (1.02–1.08) | <0.001 | 598 | RR 1.04 (1.02–1.06) | <0.001 |
| 48-h landmark cohort | Per 2-unit increase in cumulative RBC dose over the first 2 ECMO days | 1,029 | RR 1.05 (1.03–1.07) | <0.001 | 554 | RR 1.03 (1.01–1.05) | 0.005 |
| Excluding postcardiotomy / HT / LVAD | Per 2-unit increase in RBC dose on ECMO day 1 | 989 | RR 1.05 (1.02–1.08) | 0.003 | 531 | RR 1.03 (1.01–1.05) | <0.001 |
| Excluding ECPR | Per 2-unit increase in RBC dose on ECMO day 1 | 744 | RR 1.06 (1.02–1.10) | 0.007 | 323 | RR 1.05 (1.02–1.09) | 0.004 |
| Non-surgical VA-ECMO only | Per 2-unit increase in RBC dose on ECMO day 1 | 460 | RR 1.07 (1.03–1.10) | <0.001 | 195 | Not estimable (n=195) | - |
| Center-adjusted mixed-effects model | Per 2-unit increase in RBC dose on ECMO day 1 | 1,110 | OR 1.19 (1.10–1.28) | <0.001 | 598 | OR 1.18 (1.08–1.29) | <0.001 |

RBC, red blood cell; RR, risk ratio; OR, odds ratio; CI, confidence interval; ECMO, extracorporeal membrane oxygenation; VA, veno-arterial; ECPR, extracorporeal cardiopulmonary resuscitation; HT, heart transplantation; LVAD, left ventricular assist device.

* The SOFA-complete cohort included patients with complete data for the original prespecified covariates and pre-ECMO SOFA score. Patients not included in the SOFA-complete cohort had missing data in at least one original covariate and/or SOFA score; missing covariates were imputed using multiple imputation, and estimates were pooled using Rubin’s rules. Risk ratios were estimated using modified Poisson regression with robust variance estimation and adjusted for age, sex, body mass index, pre-ECMO hemoglobin, platelet count, creatinine, lactate, ECPR status, postcardiotomy status, and SOFA score. In the analysis excluding ECPR, the ECPR variable was omitted from the adjustment set. The center-adjusted model included center as a random effect and is presented as an odds ratio. The non-surgical VA-ECMO analysis in the non–SOFA-complete cohort with MI was not estimable because of the small sample size.
