# Supplementary material for: Early red blood cell transfusion burden, hemoglobin at first transfusion, and in-hospital mortality in patients supported with veno-arterial ECMO: a multicenter cohort study
Source: Ann Intensive Care. 2026 Jul 25;16:100125. doi: 10.1016/j.aicoj.2026.100125 (PMC13452410; doi:10.1016/j.aicoj.2026.100125)
Supplement: Supplementary file 4 [file mmc4.docx]

**Supplementary Table 4-A. Sensitivity analyses in consistent-threshold patients receiving ≥2 RBC units**

| Sensitivity analysis | Exposure metric | Effect estimate | P value |
| --- | --- | --- | --- |
| Primary model: 24-h landmark cohort (n= 469) | Per 2-unit increase in RBC dose on ECMO day 1 | RR 1.08 (1.04–1.12) | <0.001 |
| 48-h landmark cohort (n= 432) | Per 2-unit increase in cumulative RBC dose over the first 2 ECMO days | RR 1.05 (1.03–1.08) | <0.001 |
| Excluding postcardiotomy / HT / LVAD (n= 406) | Per 2-unit increase in RBC dose on ECMO day 1 | RR 1.06 (1.01–1.10) | 0.011 |
| Excluding ECPR (n= 271) | Per 2-unit increase in RBC dose on ECMO day 1 | RR 1.13 (1.08–1.18) | <0.001 |
| Non-surgical VA-ECMO only (n= 217) | Per 2-unit increase in RBC dose on ECMO day 1 | RR 1.05 (1.00–1.11) | 0.065 |
| Center-adjusted mixed-effects model (n= 469) | Per 2-unit increase in RBC dose on ECMO day 1 | OR 1.29 (1.16–1.45) | <0.001 |

RBC, red blood cell; RR, risk ratio; OR, odds ratio; CI, confidence interval; ECMO, extracorporeal membrane oxygenation; VA, veno-arterial; ECPR, extracorporeal cardiopulmonary resuscitation; HT, heart transplantation; LVAD, left ventricular assist device.

* This sensitivity analysis was restricted to complete-case patients in the consistent-threshold group who received ≥2 RBC units during the entire ECMO course. Because exact transfusion timestamps were not available, transfusion exposure was analyzed using ECMO day-based aggregation. RBC dose on ECMO day 1 was defined as the total number of RBC units administered on the first ECMO day, and cumulative RBC dose over the first 2 ECMO days was defined as the total number of RBC units administered across ECMO days 1 and 2. The 48-h landmark cohort additionally excluded patients who died between 24 and 48 hours after ECMO initiation. Risk ratios (RR) were estimated using modified Poisson regression models with robust variance estimation, adjusted for age, sex, body mass index, pre-ECMO hemoglobin, platelet count, lactate, creatinine, ECPR, and postcardiotomy status. In the analysis excluding ECPR, the ECPR variable was omitted from the adjustment set. The center-adjusted mixed-effects model was performed using a mixed-effects logistic regression model with center included as a random effect, and the results are presented as odds ratios (OR) with 95% confidence intervals.

**Table 4-B. Association between first-transfusion hemoglobin and in-hospital mortality in consistent-threshold patients receiving ≥2 RBC units**

| First-transfusion Hb in consistent-threshold group (n=469) | Patients | Deaths / N (%) | Adjusted RR (95% CI) | P-value |
| --- | --- | --- | --- | --- |
| <7.0 g/dL | 118 | 82/118 (69.5) | 1.25 (1.02–1.60) | 0.039 |
| 7.0–<8.0 g/dL | 96 | 59/96 (61.5) | 1.10 (0.87–1.40) | 0.435 |
| 8.0–<9.0 g/dL | 84 | 47/84 (56.0) | Reference |  |
| 9.0–<10.0 g/dL | 50 | 26/50 (52.0) | 0.96 (0.67–1.32) | 0.794 |
| ≥10.0 g/dL | 121 | 47/121 (38.8) | 0.71 (0.53–0.94) | 0.024 |
| Per 1 g/dL decrease |  |  | 1.14 (1.08–1.21) | <0.001 |
| Nonlinearity (spline) |  |  | P=0.804 |  |

Hb, hemoglobin; RR, risk ratio; CI, confidence interval

* Values are presented as number of patients, deaths/total (%), and adjusted risk ratio (RR) with 95% confidence interval (CI). The analysis was restricted to complete-case patients in the consistent-threshold group who received ≥2 RBC units during the entire ECMO course. The reference hemoglobin category was 8.0–<9.0 g/dL. Models were adjusted for age, sex, body mass index, platelet count, lactate, creatinine, ECPR, and postcardiotomy status; pre-ECMO hemoglobin was excluded from adjustment. Confidence intervals and P values were estimated using 2,000 bootstrap resamples. The per-1 g/dL decrease estimate represents an overall linear trend across hemoglobin categories, not a direct comparison between adjacent categories. Nonlinearity was assessed using a spline likelihood-ratio test.
