# Supplementary material for: Early red blood cell transfusion burden, hemoglobin at first transfusion, and in-hospital mortality in patients supported with veno-arterial ECMO: a multicenter cohort study
Source: Ann Intensive Care. 2026 Jul 25;16:100125. doi: 10.1016/j.aicoj.2026.100125 (PMC13452410; doi:10.1016/j.aicoj.2026.100125)
Supplement: Supplementary file 5 [file mmc5.docx]

**Supplementary Table 5-A. Multiplicative interaction between ECMO day-1 RBC burden and first-transfusion hemoglobin**

| First-transfusion Hb in qualifying pretransfusion group | Patients | Interaction term | Adjusted RR for interaction (95% CI) | P for interaction |
| --- | --- | --- | --- | --- |
| Complete-case transfusion cohort | 1,043 | Day-1 RBC dose per 2 units × first-transfusion Hb  per 1 g/dL decrease | 0.96 (0.94–0.98) | <0.001 |

Hb, hemoglobin; RR, risk ratio; CI, confidence interval

**Supplementary Table 5-B. Stratified association between ECMO day-1 RBC burden and in-hospital mortality according to first-transfusion hemoglobin category**

| First-transfusion Hb in qualifying pretransfusion group (n=1,043) | Patients | Deaths / N (%) | Adjusted RR per 2-unit increase  (95% CI) | P-value |
| --- | --- | --- | --- | --- |
| <7.0 g/dL | 251 | 169/251 (67.3) | 1.02 (0.98–1.06) | 0.311 |
| 7.0–<8.0 g/dL | 242 | 138/242 (57.0) | 1.06 (0.98–1.15) | 0.144 |
| 8.0–<9.0 g/dL | 218 | 121/218 (55.5) | 1.09 (1.04–1.16) | 0.001 |
| 9.0–<10.0 g/dL | 126 | 55/126 (43.7) | 1.28 (1.17–1.41) | <0.001 |
| ≥10.0 g/dL | 206 | 70/206 (34.0) | 1.18 (1.10–1.26) | <0.001 |

Hb, hemoglobin; RR, risk ratio; CI, confidence interval

* Values are presented as adjusted RRs with 95% CIs. The interaction analysis evaluated the multiplicative interaction between ECMO day-1 RBC dose, modeled per 2-unit increase, and first-transfusion hemoglobin, modeled per 1 g/dL decrease. Stratified models were fitted within each first-transfusion hemoglobin category. All models were adjusted for age, sex, body mass index, platelet count, lactate, creatinine, ECPR, and postcardiotomy status using modified Poisson regression with robust variance estimation. These analyses were exploratory.
