# Supplementary material for: Early red blood cell transfusion burden, hemoglobin at first transfusion, and in-hospital mortality in patients supported with veno-arterial ECMO: a multicenter cohort study
Source: Ann Intensive Care. 2026 Jul 25;16:100125. doi: 10.1016/j.aicoj.2026.100125 (PMC13452410; doi:10.1016/j.aicoj.2026.100125)
Supplement: Supplementary file 6 [file mmc6.docx]

**Supplementary Table 6. Fine–Gray competing-risk sensitivity analyses of day-1 RBC dose**

| *Exposure* | *N* | *In-hospital death, n* | *Discharge alive, n* | *sHR* | *95% CI* | *P-value* |
| --- | --- | --- | --- | --- | --- | --- |
| *Continuous model* | | | | | | |
| Day-1 RBC dose, per 2-unit increase | 1,140 | 595 | 545 | 1.16 | 1.11–1.21 | <0.001 |
| *Categorical model* | | | | | | |
| 0 units | 500 | 211 | 289 | Reference |  |  |
| 1–2 units | 278 | 145 | 133 | 1.28 | 1.04–1.61 | 0.026 |
| 3–4 units | 185 | 117 | 68 | 1.70 | 1.33–2.16 | <0.001 |
| ≥5 units | 177 | 122 | 55 | 1.92 | 1.51–2.45 | <0.001 |

RBC, red blood cell; ECMO, extracorporeal membrane oxygenation; sHR, subdistribution hazard ratio; CI, confidence interval; ECPR, extracorporeal cardiopulmonary resuscitation.

* Values are presented as subdistribution hazard ratios (sHRs) with 95% confidence intervals (CIs) and P-values from separate Fine–Gray competing-risk models. The event of interest was in-hospital death, and discharge alive was treated as the competing event. Time was measured from the 24-hour landmark after ECMO initiation. Day-1 RBC dose was modeled continuously per 2-unit increase and categorically, with 0 units as the reference group. Both models were adjusted for age, sex, body mass index, pre-ECMO hemoglobin, platelet count, lactate, creatinine, ECPR, and postcardiotomy status. The 95% CIs were estimated using 2,000 patient-level bootstrap resamples, and P-values were calculated from model-based standard errors. Analyses were restricted to the complete-case cohort of 1,140 patients from the 24-hour landmark cohort of 1,708 patients to maintain consistency with the primary adjusted analysis.
