# Supplementary material for: Early red blood cell transfusion burden, hemoglobin at first transfusion, and in-hospital mortality in patients supported with veno-arterial ECMO: a multicenter cohort study
Source: Ann Intensive Care. 2026 Jul 25;16:100125. doi: 10.1016/j.aicoj.2026.100125 (PMC13452410; doi:10.1016/j.aicoj.2026.100125)
Supplement: Supplementary file 7 [file mmc7.docx]

**Supplementary Table 7. Exploratory day-5 landmark analysis of late RBC transfusion burden and in-hospital mortality**

| Analysis model | Analysis population | Exposure metric | N | Death / N (%) | Adjustment | RR (95% CI) | P-value |
| --- | --- | --- | --- | --- | --- | --- | --- |
| Fully adjusted model | Day-5 landmark cohort with complete covariate data | ECMO days 3–5 cumulative RBC units, per 2-unit increase | 659 | 359/659 (54.5) | Early RBC burden  + Covariates | 1.10 (1.05–1.14) | <0.001 |
| Early-RBC adjusted sensitivity model | Overall day-5 landmark cohort | ECMO days 3–5 cumulative RBC units, per 2-unit increase | 957 | 509/957 (53.2) | Early RBC burden only | 1.09 (1.06–1.13) | <0.001 |

RBC, red blood cell; RR, risk ratio; OR, odds ratio; CI, confidence interval; ECMO, extracorporeal membrane oxygenation; VA, veno-arterial; ECPR, extracorporeal cardiopulmonary resuscitation; HT, heart transplantation; LVAD, left ventricular assist device.

* Values are presented as risk ratios (RR) with 95% confidence intervals (CI). The day-5 landmark cohort was defined as patients with ECMO duration ≥120 hours. Late RBC burden was defined as the cumulative number of RBC units administered across ECMO days 3–5. Early RBC burden was defined as the cumulative number of RBC units administered across ECMO days 1 and 2. RBC burden variables were divided by 2 before modeling so that effect estimates represent the RR per 2-unit increase in RBC transfusion. The complete-covariate analysis population was defined as patients in the day-5 landmark cohort with non-missing data for all variables included in the fully adjusted model. Patients with zero RBC transfusion during ECMO days 3–5 were retained in the analysis. Covariates included Age, Sex, BMI, Pre-ECMO hemoglobin, Platelet count, Creatinine, Lactate, ECPR, and Postcardiotomy. Modified Poisson regression with robust variance estimation was used, and 95% CIs were obtained from 2,000 bootstrap resamples.
